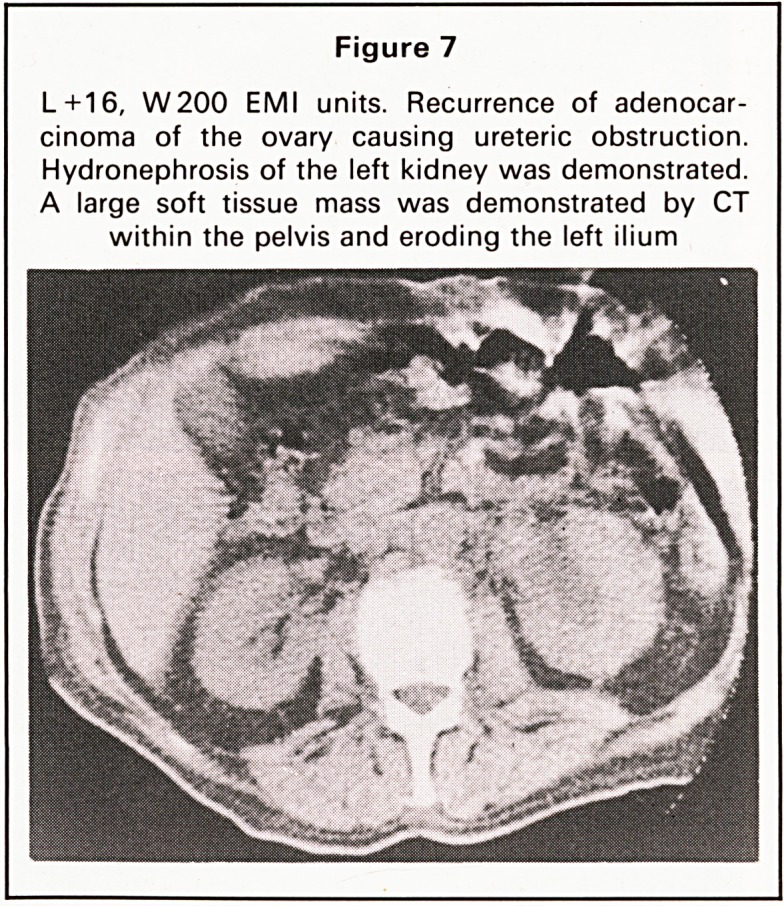# The Radiology of Tumours of the Pelvis

**Published:** 1984-10

**Authors:** Paul Goddard, Sally Scott, E. Rhys Davies

**Affiliations:** The Imaging Research Unit, Bristol Royal Infirmary; The Imaging Research Unit, Bristol Royal Infirmary; The Imaging Research Unit, Bristol Royal Infirmary

## Abstract

The radiology of tumours involving the bony pelvis was reviewed in 35 patients. In all of the patients computed tomography (CT) and plain radiography had been done and histological confirmation of the diagnosis obtained. In 12 patients radio-isotope skeletal scanning had been done also.

CT was invaluable for demonstrating the intraosseous extent of tumours and for giving additional information about the adjacent soft tissue involvement. In some cases, lesions distant from the primary site of interest were displayed on CT. This was particularly valuable where there were lymph node deposits or multiple skeletal deposits. In one case unsuspected hydro-nephrosis was shown.

The cause of false positive results in the sacrum was also studied.


### The Radiology of Tumours of the Pelvis

**Paul Goddard, Sally Scott* and E. Rhys Davies**
The Imaging Research Unit, Bristol Royal Infirmary

### SUMMARY

The radiology of tumours involving the bony pelvis was reviewed in 35 patients. In all of the patients computed tomography (CT) and plain radiography had been done and histological confirmation of the diagnosis obtained. In 12 patients radio-isotope skeletal scanning had been done also.

CT was invaluable for demonstrating the intra-osseous extent of tumours and for giving additional information about the adjacent soft tissue involvement. In some cases, lesions distant from the primary site of interest were displayed on CT. This was particularly valuable where there were lymph node deposits or multiple skeletal deposits. In one case unsuspected hydro-nephrosis was shown.

The cause of false positive results in the sacrum was also studied.

### INTRODUCTION

Osteolytic lesions in the bony pelvis can prove difficult to detect on plain radiography for a number of well-recognised reasons. Thus, overlying bowel gas may mimic a bony lesion, or the angulation of the sacrum may make it difficult to determine its outline clearly. Even when a lesion is detected the full extent of its extra-osseous component is not shown unless it is very large, and this is an important shortcoming in planning a field of radiotherapy treatment.

Computed tomography is a well-known method of studying tumours of the soft tissue, but skeletal involvement is usually recorded incidentally. Indeed, apart from bone density assessment, skeletal CT is undervalued despite its good demonstration of the bony pelvis.[1,2] The role of CT in the radiological investigation of pelvic bone tumours and the demonstration of neoplastic deposits has been studied, and a phantom study done to determine the effect of scanning the pelvis at varying angles of tilt.

### MATERIALS AND METHODS

(a) *Phantom.* Experiments were performed to determine how the position of the pelvis affected the CT image. A standard Rando phantom was scanned with the pelvis at varying angles to the trans-axial plane. The phantom was aligned in three positions (Figure 1):

**Figure 1**

The sacrum of a Rando man phantom was scanned at various angles

(a) Lying flat on the back.
(b) Head raised 30°.
(c) Caudally raised 25°.

The phantom was radiographed and supine angled cone view of the sacrum and lateral views were obtained.

(b) *Clinical.* Thirty-five patients presenting to the Bristol Radiotherapy Centre, 1977 to 1981 were referred for CT with suspected primary or secondary neoplastic lesions of the pelvic bones (Table 1). All had plain radiography of the pelvis and 12 also had $^{99}Tc^{m}$-methylene disphosphonate skeletal scans. The initial reports were studied and all the investigations were reviewed, retrospectively. In all positive investigations, clinical and pathological correlation was obtained. In particular, it was noted

* Present address: Department of Radiodiagnosis, Southampton General Hospital.

**Table 1**

| | No. of cases | Age range at time of investigation (years) |
|---|---|---|
| Carcinoma of rectum | 8 | 40–68 |
| Carcinoma of cervix | 7 | 56–71 |
| Carcinoma of breast | 4 | 42–55 |
| Lymphoma and lymphosarcoma | 4 | 37–70 |
| Carcinoma of prostate | 3 | 64–71 |
| Sarcoma (soft tissue) | 2 | 46–62 |
| Ewing's | 2 | 14–30 |
| Myeloma | 1 | 48 |
| Adenocarcinoma of ovary | 1 | 35 |
| Rhabdomyosarcoma | 1 | |
| Carcinoma of thyroid | 1 | 65 |
| Teratoma | 1 | |

Thirty-five cases – age range 14–71 years.

whether or not the CT provided more information, the same information or less information than other radiological investigations. Discrepancies between the original report and the retrospective opinion were noted.

### RESULTS

### PHANTOM EXPERIMENTS

The CT scans of the Rando man phantom showed clearly that angling the pelvis resulted in a considerable alteration in the resolution and thickness of the bony cortex. Due to desiccation, the medullary bone of the phantom was absent and the skeleton contained air. Even allowing for this it was apparent that by changing the angulation of the pelvis the sacrum and ilium appear to be eroded whilst being shown as intact in other positions (Figure 2).

(a)

(b)

(c)

**Figure 2**

Scans of the Rando phantom. (a) Lying flat. (b) Head raised. (c) Caudally raised. Due to desiccation the medullary bone of the phantom is absent and the skeleton contains air. Even allowing for this it is apparent that by changing the angle of the pelvis there is considerable alteration in the appearance of the sacrum. Raising the phantom caudally (c) gave the most easily interpretable image

### RADIOGRAPHY COMPARED WITH CT

The neoplastic conditions studied are tabulated (Table 1).

CT gave the same information as plain radiography in nine cases. In four cases the sacral defect had been reported initially (Figure 3) but review, after the phantom experiment had been done, indicated clearly that these appearances were normal. The original reports were falsely positive.

CT gave more information than plain radiography in all of the remaining 26 cases (Table 2).

In one case a large soft tissue mass was correctly reported but erosion of the ilium was missed. The erosion was detected on review but represents one 'false negative result' on reporting.

These findings are summarised in Table 2.

**Table 2**

**Review of CT and X-rays**

| | |
|---|---|
| Identical information CT and radiographs (reported false positives on CT: 4) | 9 |
| Greater information on CT than on radiographs (reported false negative: 1) | 26 |
| Less information on CT than X-rays of pelvis | 0 |

### $^{99}Tc^{m}$-MDP SCANS COMPARED WITH CT

Twelve patients had radio-isotope bone scans. On review there was agreement on the presence or absence of pelvic bone lesions in nine cases. In one of these nine, the bone scan was correctly reported as normal but CT provided a reported false positive result. In another three of these cases the bone scans showed unsuspected distant metastases. In two cases bone scans were normal but CT correctly showed pelvic lesions and in the final case bone scan showed only one lesion but multiple metastases were detected by CT.

### CHEST RADIOGRAPHY

One patient had pulmonary metastases shown on a chest X-ray immediately after the CT scan. At least four cases had pulmonary metastases within 6 months.

### CT APPEARANCES OF THE NEOPLASTIC PROCESSES

A variety of neoplastic processes was studied (Table 1), and the following CT signs were elicited:

*Destruction*. Osteolytic lesions were clearly demonstrated as areas of low attenuation value compared with surrounding bone (see Figure 4). The lesions were asymmetrically positioned.

**Figure 3**

(L 24) (W 400) A 'false' position finding (normal variant). There is an apparent defect asymmetrically in the anterior border of the sacrum in (a). On the scan (b) performed immediately craniad the defect is 'filled in' but the patterns of the ala are asymmetrical. The cause of the asymmetry is shown on scan (c) (13 mm craniad to (b)) to be the partial sacralisation of L5. (The right transverse process of L5 is present articulating with the ilium.)

(a)

(b)

(c)

(a)

**Figure 4**

(L +11) (W 200) – myeloma. (a) Lower sacrum. (b) Mid sacrum. (c) Upper sacrum. Scans (a), (b) and (c) showing asymmetrical destruction of the sacrum due to myeloma with the cortex breached anteriorly on the left side. Unlike Figure 1 the defect cannot be completed by the scans above or below

(b)

(c)

(a)

**Figure 5**

Ewing's (a) Scan at the anatomical level of the superior pubic rami. (L 60, W 400). (b) Scan 5 cm above (a). (c) Line drawing of (b). CT scan (a) reveals a patchy lytic lesion in the right superior pubic ramus with expansion of bone and destruction of cortex. There is swelling of the adjacent soft tissues. The scans craniad (b) showed compression of the anterior wall of the bladder. At surgery the tumour was adherent to the bladder wall but did not invade it

(b)

(c)

**Figure 6**

Thyroid carcinoma metastatic deposit. (a) Plain radiograph of the pelvis. (b) CT scan (L9, W200). The plain radiograph of the pelvis revealed an osteolytic lesion of the right ilium with expansion, an incomplete bony rim and soft tissue swelling. The CT scan clearly demonstrated the soft tissue swelling and displacement of muscle planes but no additional information was provided

(a)

(b)

**Figure 7**

L+16, W200 EMI units. Recurrence of adenocarcinoma of the ovary causing ureteric obstruction. Hydronephrosis of the left kidney was demonstrated. A large soft tissue mass was demonstrated by CT within the pelvis and eroding the left ilium

*Sclerosis.* Endosseous margins were clearly seen and in some cases sclerosis was present.

*Fracture.* Discontinuity of the cortex and pathological fractures were also seen (Figure 4).

*Soft tissue extension.* Local soft tissue extension was present in 18 cases. This was usually displayed as a large soft tissue mass displacing (Figure 6b) or obliterating the outline of surrounding muscular or other structures (Figure 5a). In one case of Ewing's sarcoma the soft tissue mass was shown to indent the bladder (Figure 5b). At surgery the tumour was adherent to the bladder surface and was compressing the bladder but did not invade the wall.

*Distant lesions.* In two cases lymph node enlargement was shown and in one case hydronephrosis was present (Figure 7).

### DISCUSSION

The phantom experiments show that variations in the angle of the sacrum alter its configuration on tomography, leading to a misleading appearance of its anterior margin. This effect can be overcome if the CT scanner gantry can be angled in line with the sacrum. This is not possible with all models and if there appears to be an anterior sacral defect, the scan immediately above must be studied carefully to see if the presumed defect is immediately adjacent to the sacral promontory (Figure 3). If the patient is positioned correctly the false defect will be central and will correspond to the sacral outline on the adjacent scan. Symmetry is an important index of normality. Defects that are asymmetrical or non-central and extend to adjacent scans are true positives. In one instance partial sacralisation of the fifth lumbar vertebra led to a misleading appearance but the reality was apparent on the plain film, thereby emphasising the vital need to link CT with standard radiographs at the time of reporting. The nature of the analysis was such that the absolute sensitivity of CT in showing lesions could not be assessed accurately but it is notable that the only misleading appearances were those that led to false recording of sacral lesions.

All the radiographic lesions were detected by CT

and in 26 of the 35 cases, CT provided new information. Most of this information was related to the extra-osseous spread of lesions. This is notoriously difficult to detect by plain radiography even when the soft tissue component of the lesion is so large that the function of adjacent organs such as bladder and ureter is compromised.[3–7] Contrast studies of the gut and urinary tract may of course supplement plain radiography but CT often provides adequate information without contrast enhancement. The obligatory imaging of all structures within the section is an important advantage of CT, e.g. when unexpected lesions are shown in lymph nodes[8,9] or elsewhere. However, the main advantage is in determining the precise extent of lesions so that the tumour volume can be calculated for the purpose of treatment planning.[4,7,9,10]

The only disappointments of CT of the pelvis are that the absorption coefficient of tumours is unrelated to their cellular type and do not help to discriminate between the various kinds of tumour.[9] In general the quality of bone response is limited to a positive or negative recording, but the recognition of bone erosion considerably raises the certainty of malignancy.

It is impracticable to view the whole skeleton by CT in every case, and the most effective way of doing this is by $^{99}Tc^{m}$-methylene disphosphonate skeletal survey.[11] The number and site of distant lesions can be shown well by this technique which has a high sensitivity for skeletal abnormalities of all kinds.[11,12] However, the radioactive marker is often incorporated into bone adjacent to tumour, and not into tumours themselves. Thus it overestimates the extent of lesions and may give rise to false positives and false negatives so that it has a low specificity.[11,12] Further, extra-osseous extension is not shown unless the tumour has calcified or is forming bone.

Sclerotic lesions are shown particularly well by CT and by conventional radiography. It is unlikely that they are an indication for CT unless there is evidence of a breach of bone cortex.[13] Paget's disease is among the important causes for dense bone in the age group that has a high incidence of bone metastases and it is important to note that the distinction is more easily made by plain radiographs.

In summary, CT is very valuable in the local assessment of osteolytic lesions of the bony pelvic. The full extent of the lesion is shown and unexpected adjacent lesions are detected. Radionuclide bone scanning remains the method of choice for detecting distant skeletal abnormalities.

### ACKNOWLEDGEMENTS

The authors are indebted to Miss J. Hugh (typing) and Mr. E. Turnbull (artwork) and the Department of Medical Illustration.

### REFERENCES

1. REDMAN, H. C. (1977) C.T. of the pelvis. *Radiol.Clin. of N.Amer.* **15**, 441–448.
2. GILULA, L. A., MURPHY, W. A., TAYLOR, C. C. and PATEL, R. B. (1979) Computed tomography of the osseous pelvis. *Radiology* **132**, 107–114.
3. WILSON, J. S., KORBOKIN, H., GENANT, H. K. and BOVILL, E. C. J. (1978) Computed tomography of musculoskeletal disorders.
4. HODSON, N. J., HUSBAND, J. E. and MACDONALD, J. S. (1979) The role of computed tomography in the staging of bladder cancer. *Clinical Radiology* **30**, 389–395.
5. LEVINE, E., KYO RAK LEE, NEFF, J. R., MAKLAD, N. F., ROBINSON, R. G. and PRESTON, D. F. (1979) Comparison of computed tomography and other imaging modalities in the evaluation of musculoskeletal tumors. *Radiology* **131**, 431–437.
6. HUSBAND, J. E., HODSON, N. T. and PARSONS, C. A. (1980) The use of computed tomography in recurrent rectal tumours. *Radiology* **134**, 677–682.
7. MORGAN, C. L., CALKINS, R. F. and CAVALCANTI, E. J. (1981) Computed tomography in the evaluation, staging and therapy of carcinoma of the bladder and prostate. *Radiology* **140**, 751–761.
8. PLAQUETTE, F. R., AHIY, A. S., CARSON, P. L., MACK, L. A., IBBOTT, G. S. and JOHNSON, M. L. (1979) A comparative study of computerized tomography and ultrasound imaging for treatment planning of prostatic carcinoma. *Co-Int.J.Radiat. Oncol.Biol.Phys.* 1979, 5/2, 289–294.
9. GORE, R. M., MOSS, A. A. and MARGULIS, A. R. (1982) The assessment of abdominal and pelvic neoplasia. The impact of C.T. In Current Problems in Surgery, Vol. XIX, No. 9. "pages 493–552, Year Book Medical Publishers Incorporated"
10. BIIZEL, H. E., LIVINGSTONE, P. A. and GRAYSON, E. V. (1979) Radiotherapeutic applications of pelvic computed tomography. *Journal of Computer Assisted Tomography* **3**, 453–466.
11. BRADY, K. W. and GOLI, H. N. (1979) The role of bone scanning in the cancer patient. *Skeletal Radiology* **3**, No. 4, 217–222.
12. MERRICK, M. V. (1975) Review article – bone scanning. *British Journal of Radiology* **48**, 327–351.
13. HOLLAND, J. F. and FREI, E. (1973) Cancer Medicine. Philadelphia, Lea & Febiger, p. 468.

## Figures and Tables

**Figure 1 f1:**
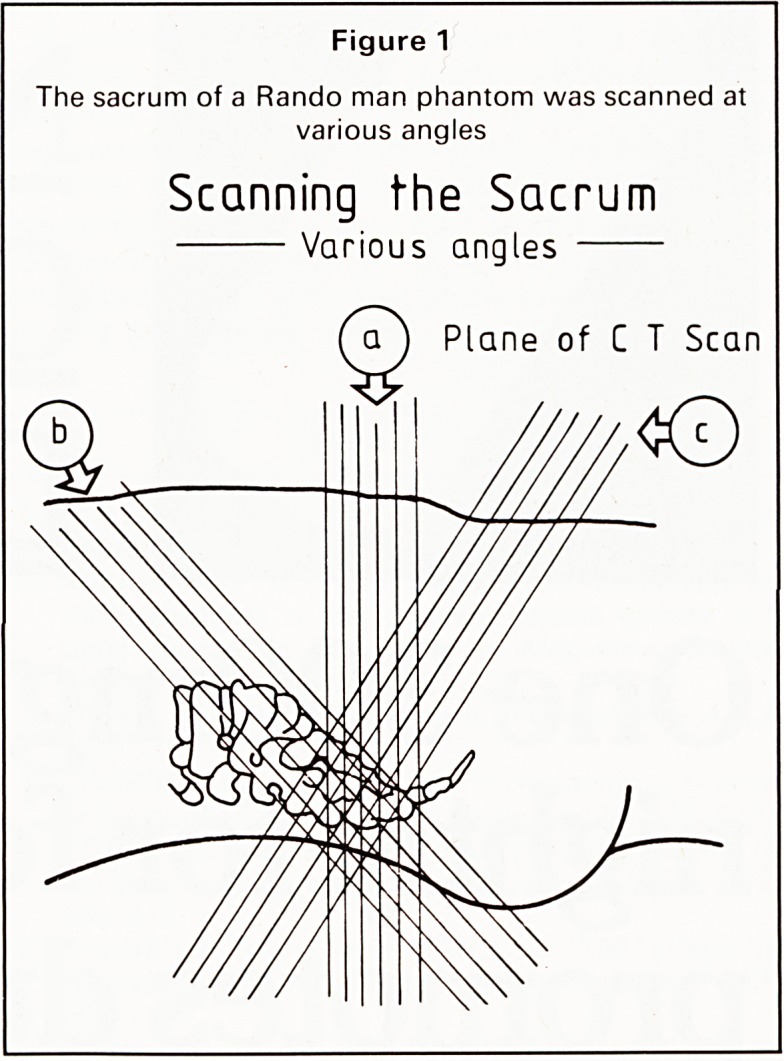


**Figure 2 f2:**
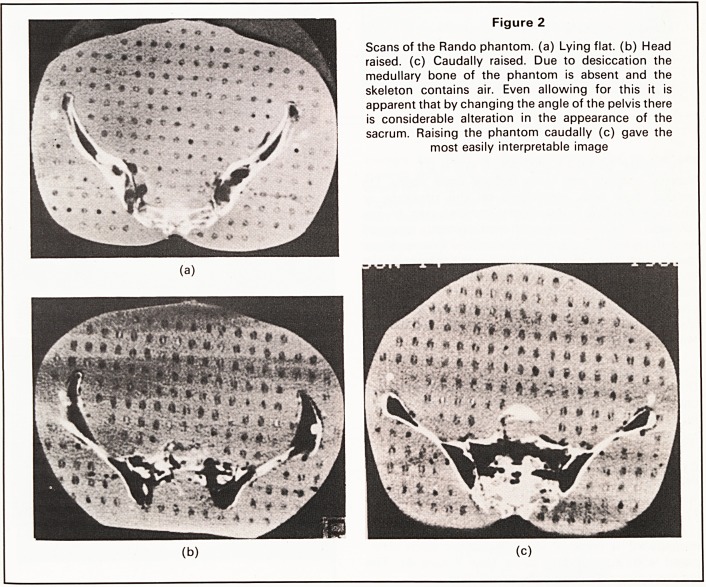


**Figure 3 f3:**
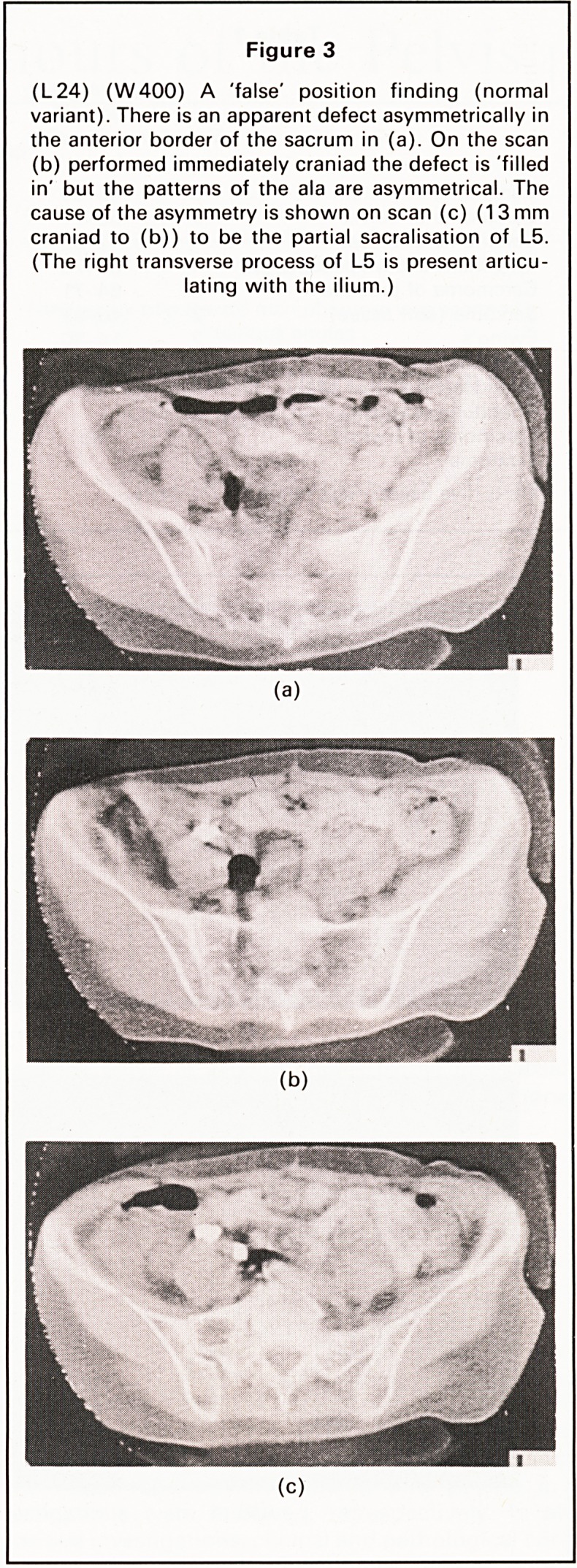


**Figure 4 f4:**
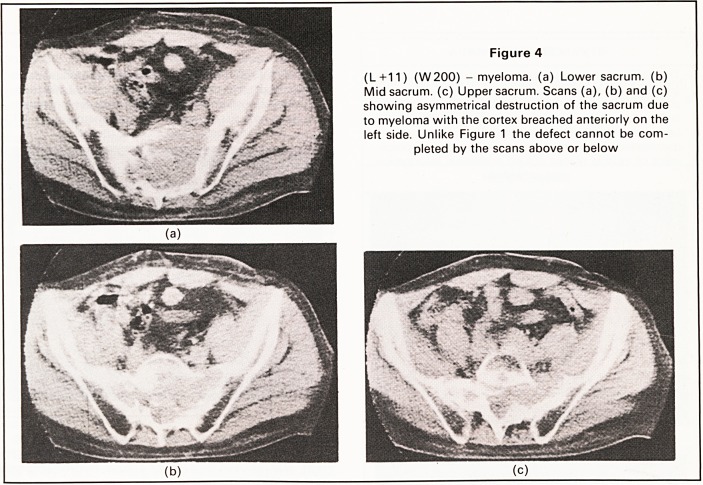


**Figure 5 f5:**
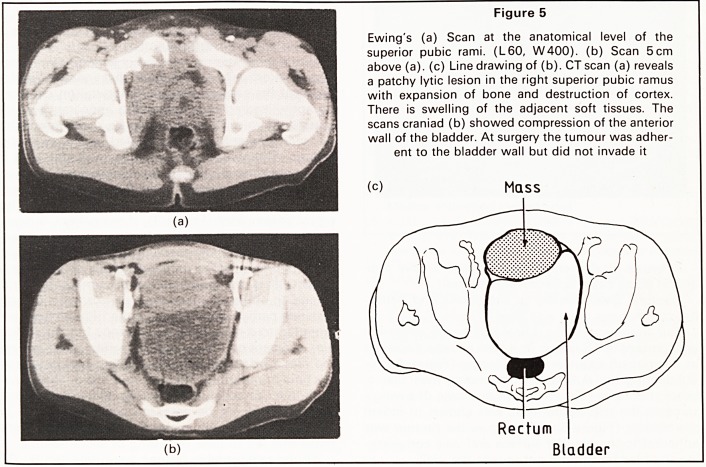


**Figure 6 f6:**
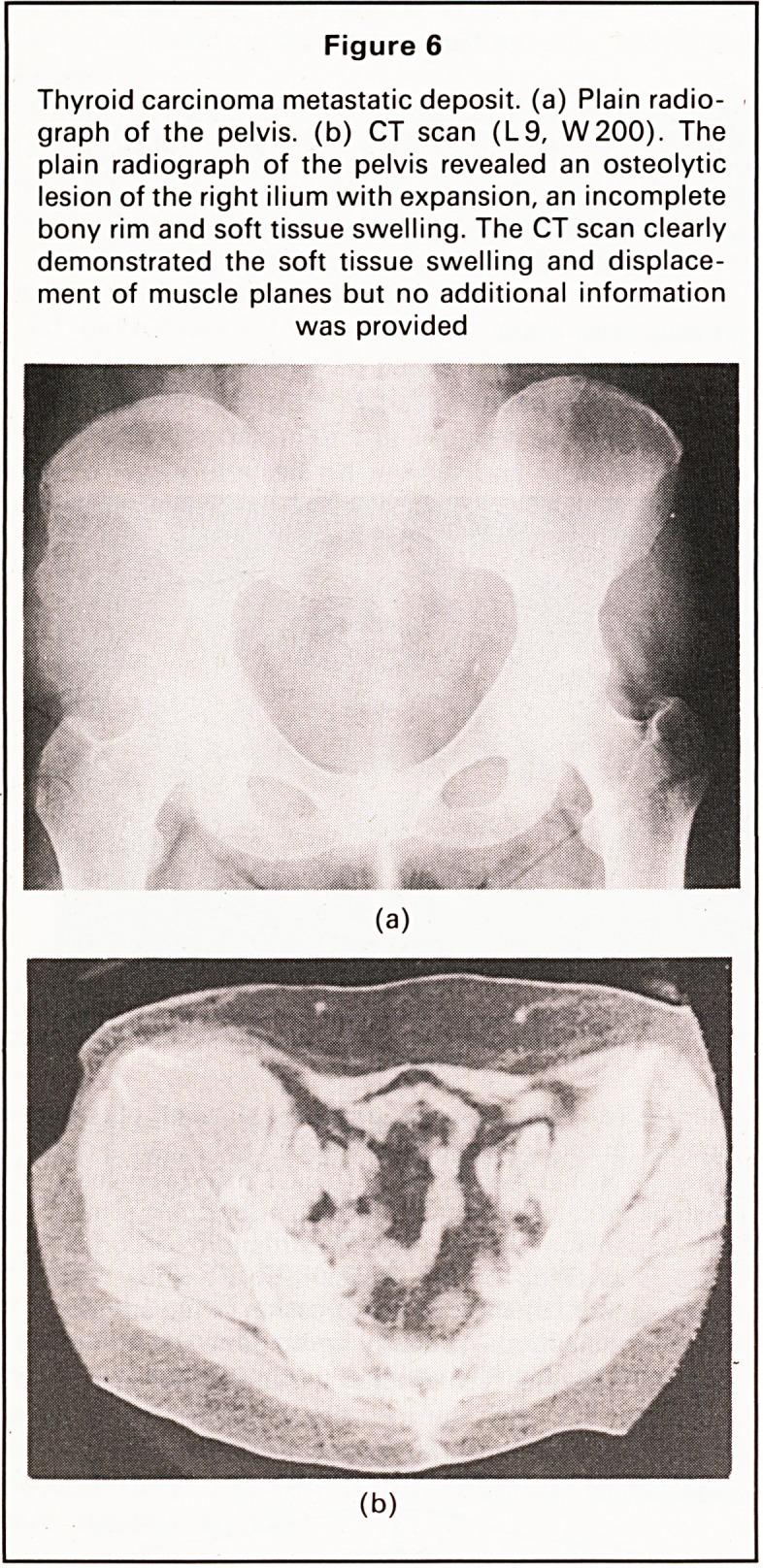


**Figure 7 f7:**